# Supplementary material for: Microwave-Assisted Synthesis of High-Energy Faceted TiO2 Nanocrystals Derived from Exfoliated Porous Metatitanic Acid Nanosheets with Improved Photocatalytic and Photovoltaic Performance
Source: Materials (Basel). 2019 Nov 4;12(21):3614. doi: 10.3390/ma12213614 (PMC6862389; doi:10.3390/ma12213614)
Supplement: Supplementary file 1 [file materials-12-03614-s001.pdf]

materials

Supplementary Materials

Microwave-Assisted Synthesis of High-Energy Faceted TiO_2 Nanocrystals Derived from Exfoliated Porous Metatitanic Acid Nanosheets with Improved Photocatalytic and Photovoltaic Performance

Yi-en Du *, Xianjun Niu, Wanxi Li, Jing An, Yufang Liu, Yongqiang Chen *, Pengfei Wang, Xiaojing Yang * and Qi Feng

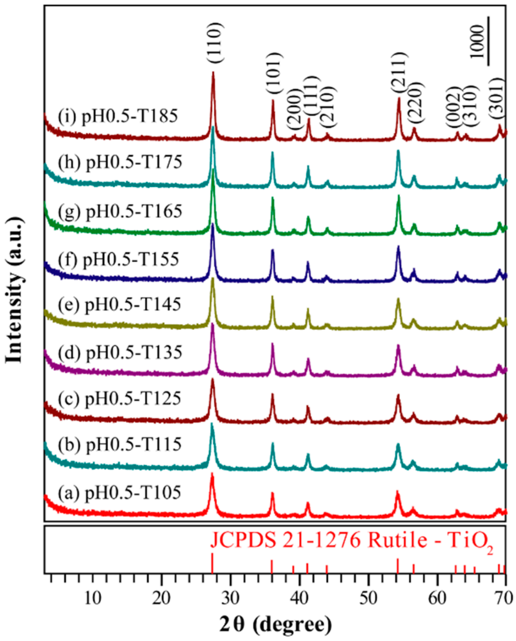

Figure S1. Evolution of the XRD patterns of the TiO_2 nanocrystals samples synthesized at pH = 0.5 and various temperatures. Evolution of the XRD patterns of the TiO_2 nanocrystals samples synthesized at pH0.5-13.5 and various temperatures; UV–vis spectral changes of MO solutions as a function of UV irradiation time in the presence of (**a**) pH4.5-T175, (**b**) pH6.5-T175, (**c**) P25 photocatalysts and (**d**) absence of photocatalyst; and particles size distributions of (**a**) pH2.5-T175, (**b**) pH4.5-T175, (**c**) pH6.5-T175, and (**d**) P25.

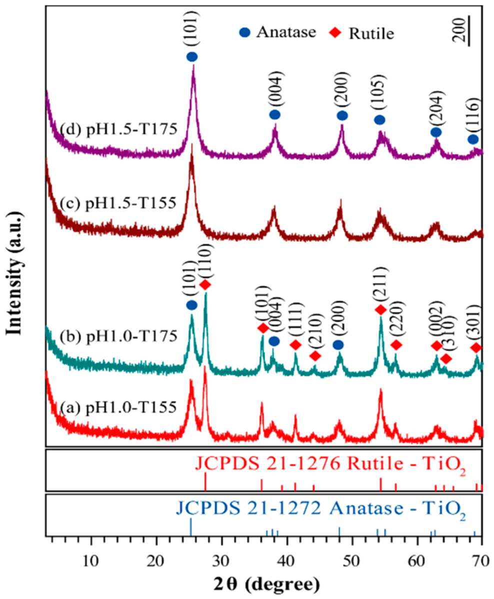

Figure S2. Evolution of the XRD patterns of the TiO_2 nanocrystals samples synthesized at pH = 0.5, 1.0, and various temperatures.

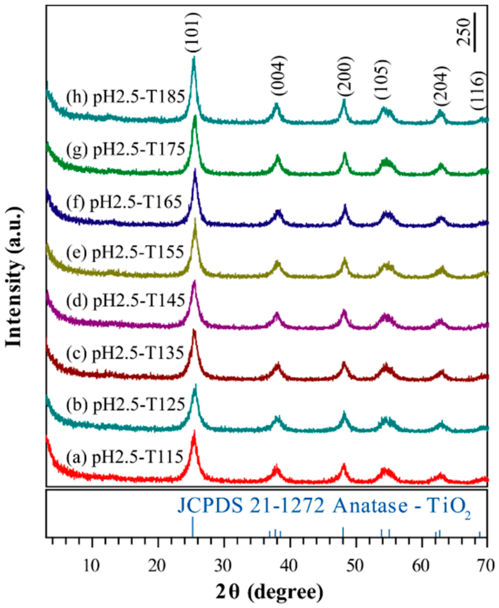

Figure S3. Evolution of the XRD patterns of the TiO_2 nanocrystals samples synthesized at pH = 2.5 and various temperatures.

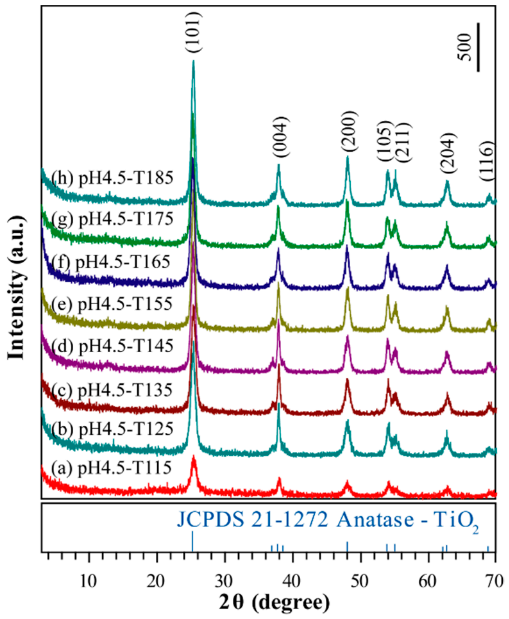

Figure S4. Evolution of the XRD patterns of the TiO_2 nanocrystals samples synthesized at pH = 4.5 and various temperatures.

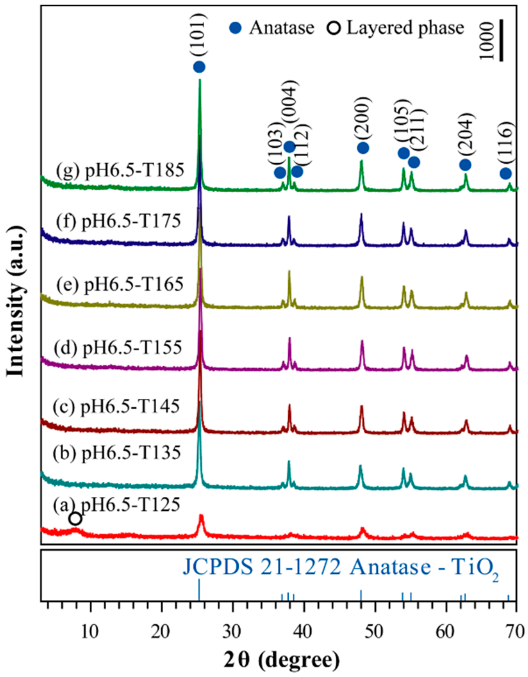

Figure S5. Evolution of the XRD patterns of the TiO_2 nanocrystals samples synthesized at pH = 6.5 and various temperatures.

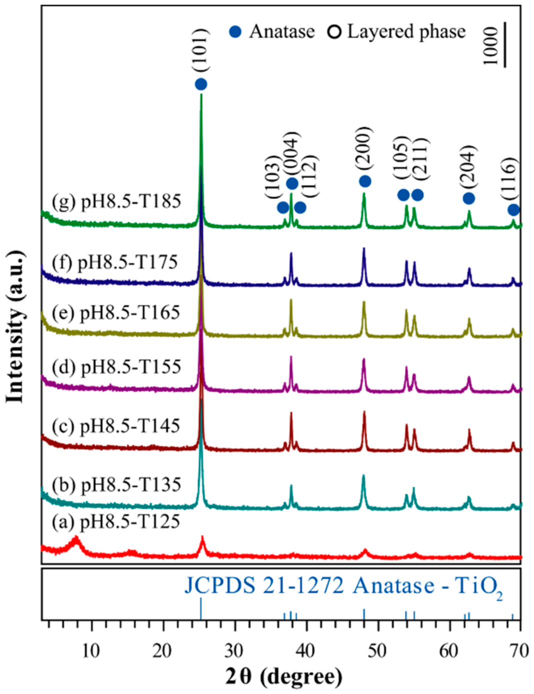

Figure S6. Evolution of the XRD patterns of the TiO_2 nanocrystals samples synthesized at pH = 8.5 and various temperatures.

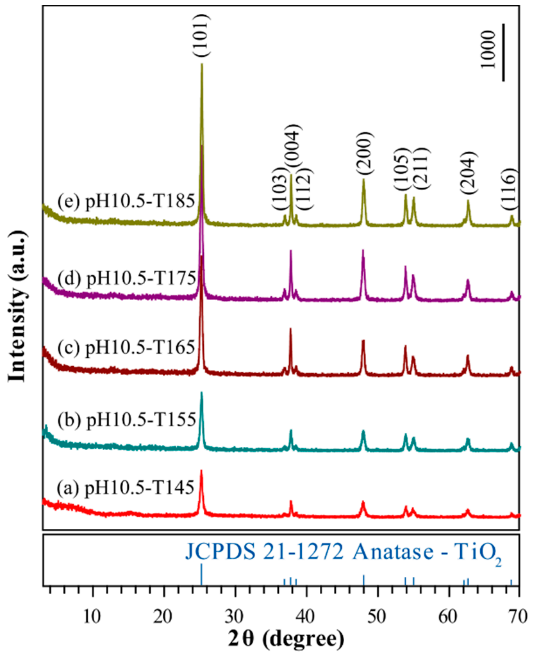

Figure S7. Evolution of the XRD patterns of the TiO_2 nanocrystals samples synthesized at pH = 10.5 and various temperatures.

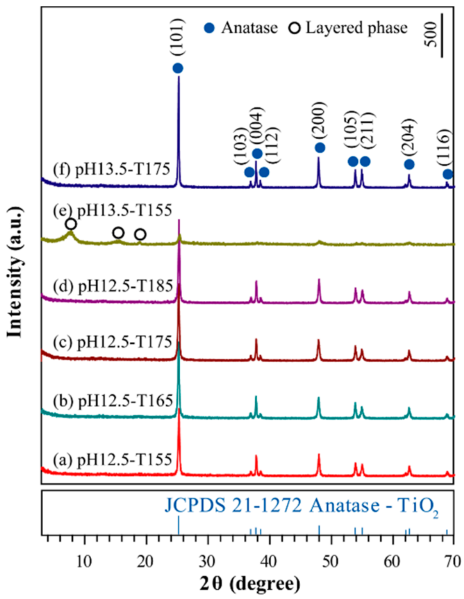

Figure S8. Evolution of the XRD patterns of the TiO_2 nanocrystals samples synthesized at pH = 12.5, 13.5 and various temperatures.

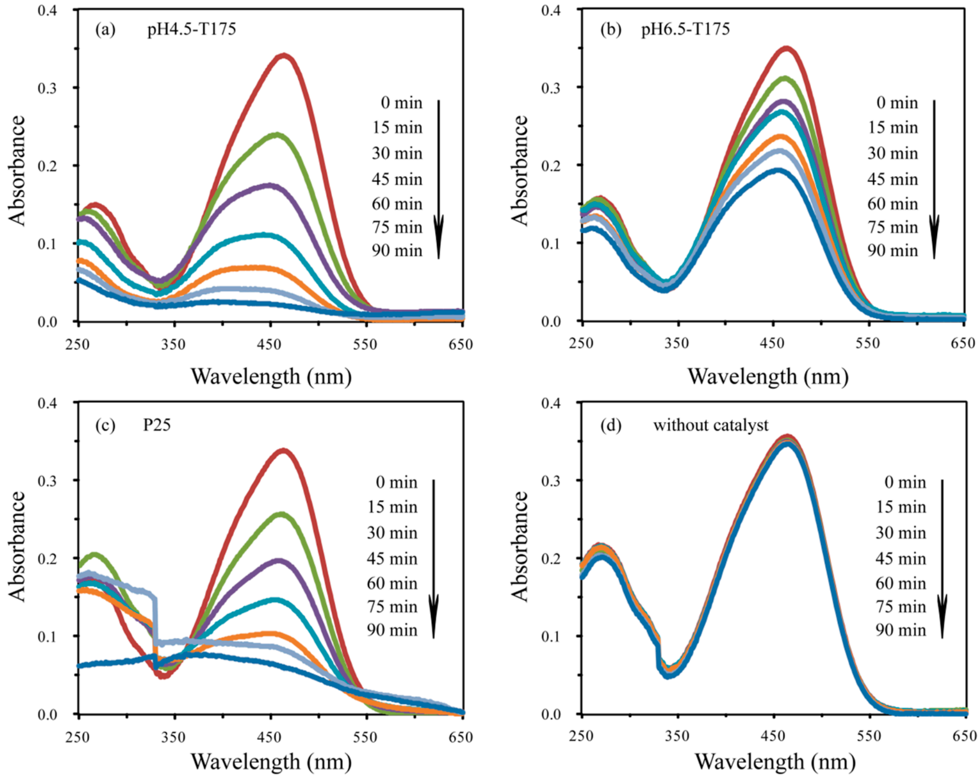

Figure S9. UV–vis spectral changes of MO solutions as a function of UV irradiation time in the presence of (**a**) pH4.5-T175, (**b**) pH6.5-T175, (**c**) P25 photocatalysts and (**d**) absence of photocatalyst.

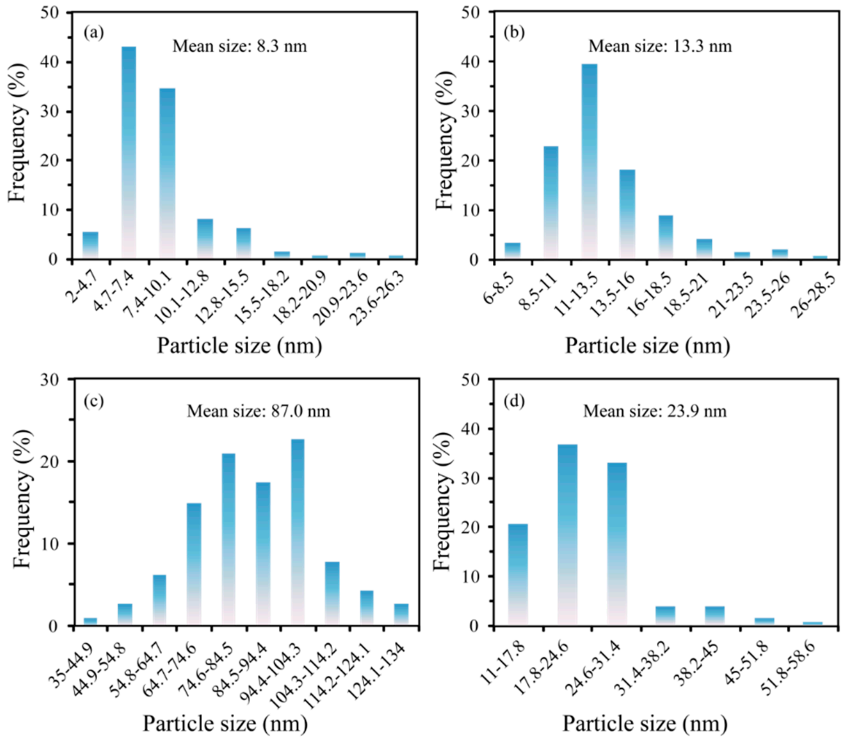

Figure S10. Particles size distributions of (**a**) pH2.5-T175, (**b**) pH4.5-T175, (**c**) pH6.5-T175, and (**d**) P25.
